# Effects of Omega-3 Polyunsaturated Fatty Acids on Heart Function and Oxidative Stress Biomarkers in Pediatric Patients with Dilated Cardiomyopathy

**Published:** 2013-03-15

**Authors:** Omidreza Firuzi, Nader Shakibazad, Hamid Amoozgar, Mohammad Borzoee, Saeed Abtahi, Gholamhossein Ajami, Pegah Ardi, Ramin Miri

**Affiliations:** 1 Medicinal and Natural Products Chemistry Research Center, Shiraz University of Medical Sciences, Shiraz, IR Iran; 2 Division of Pediatric Cardiology, Department of pediatrics, Shiraz University of Medical Sciences, Shiraz, IR Iran; 3 Cardiovascular Research Center, Shiraz University of Medical Science, Shiraz, IR Iran

**Keywords:** Dilated Cardiomyopathy, Polyunsaturated Fatty Acid, Omega-3

## Abstract

**Objectives:**

Dilated cardiomyopathy is the most prevalent type of cardiomyopathy in children, which results in congestive heart failure and causes significant morbidity and mortality. This study, aims to investigate the effect of supplementation with omega-3 polyunsaturated fatty acids (n-3 PUFA) on heart function and oxidative stress biomarkers in these patients.

**Methods:**

The present research was a case-control study on pediatric patients with dilated cardiomyopathy, who received n-3 PUFA and anti-failure therapy for 6 months (group 1, n = 6), or anti-failure therapy alone for 6 months (group 2, n = 6), as well as age matched normal individuals (group 3, n = 6), and evaluated the cardiac function and biomarkers of oxidative stress.

**Results:**

Echocardiographic parameters, such as left ventricular ejection fraction, shortening fraction, tissue Doppler Ea and Aa waves of lateral annulus of tricuspid valve, and Ea and S wave of septum, were significantly improved in group 1 after n-3 PUFA compared to pre- treatment status, while they were not changed after treatment in group 2. Antioxidant enzymes, including catalase and glutathione peroxidase activities in erythrocytes were slightly decreased, while plasma 8-iso-prostaglandin F2α concentrations were somewhat increased in group 1 compared to groups 2 and 3, however these changes were not statistically significant. Total antioxidant capacity of plasma was similar in all 3 groups.

**Conclusions:**

The results indicate that some echocardiographic parameters were significantly improved in patients receiving omega-3 fish oil. However, omega-3 had no significant effect on oxidative stress biomarkers.

## 1. Background

Dilated cardiomyopathy (DCMP) is a disorder of cardiac muscle and is characterized by a dilated ventricular chamber, mainly left ventricle and systolic dysfunction, which results in congestive heart failure (CHF) and causes significant morbidity and mortality (1-3). DCMP is the most prevalent type of cardiomyopathy in children that commonly leads to cardiac transplantation in adults and children (2,4). Towbin and co-workers have reported the incidence of DCMP in pediatric age group as 0.57 cases per 100,000 per year. They also stated that the incidence of the disorder was higher in boys than in girls and the disease was more prevalent in infants less than 1 year- old compared to the older children (4).

Primary DCMP is either acquired or occurs as a result of genetic trait. Familial type of DCMP develops in 20-50% of the cases and autosomal-dominant inheritance is more prevalent compared to X-linked, autosomal recessive, and mitochondrial inheritance. Acquired primary disease can be secondary to inflammatory diseases, such as myocarditis, or due to a large number of systemic causes (1,2). Echocardiography is an excellent non-invasive method for detecting DCMP in children (4-7). The principle in treatment of dilated cardiomyopathy is mainly directed toward reducing the heart-failure symptoms and preventing the disease progression as well as the related complications, such as stroke (2).

Reactive oxygen species including free radicals like hydroxyl radical and superoxide anion and non-radical species such as hydrogen peroxide may play a major role in oxidative stress- related disorders, including cardiovascular diseases (8,9). This has led to the hypothesis that antioxidants can be used as an efficient means of prevention and perhaps treatment of coronary artery diseases, stroke, and peripheral vascular disorders (10). Epidemiological studies support the notion that antioxidants can prevent certain diseases including cardiovascular diseases (11). Some interventional trials have failed to show beneficial effects of antioxidants, but the use of efficient antioxidants in carefully chosen patients with high levels of oxidative stress can probably provide valuable therapeutic tools (10,12).

Omega-3 polyunsaturated fatty acid (n-3 PUFA) including Eicosapentaenoic acid acid and docosahexaenoic acid have recently been used for many medical purposes and may have several applications including lowering the risk of cardiovascular diseases (13-16). The exact mechanisms, which prevent the progression of heart failure by n-3 PUFA, are not well defined. Nevertheless, suppression of inflammatory response and increased secretion of adiponectin by these antioxidant supplements could prevent the left ventricular dysfunction as well as heart failure (17-20).

Several studies have been conducted to assess the effect of n-3 PUFA on heart failure (20,21) and other heart diseases in adults (22), However, limited studies have been performed on children with heart diseases.

The aim of the present study was to determine the effect of n-3 PUFA on heart function parameters and oxidative stress biomarkers in pediatric patients with DCMP.

## 2. Materials and Methods

### 2.1 Subjects and treatments

This case-control study recruited 18 individuals from subjects, referred to Namazi hospital, affiliated to Shiraz University of Medical Sciences Shiraz, IR Iran. Three groups of the study consisted of 6 pediatric patients diagnosed with DCMP, who received n-3 PUFA and anti-failure therapy for 6 months (group 1), 6 DCMP patients receiving anti-failure therapy and vitamin (vitamin AandD) (group 2), and 6 healthy volunteers with the same age as the patients in group 1 who received only daily vitamin (group 3).

The clinical and echocardiographic features of the patients were compared in order to evaluate the effect of n-3 PUFA on the heart function of these patients. All echocardiographies were done by a pediatric cardiologist who blinded to groups and the fish oil was supplied by a nurse who was blinded to result of clinical and para-clinical data.

The study was approved by the Ethics Committee of Shiraz University of Medical Sciences, Shiraz, IR Iran and was in accordance with the guidelines of the declaration of Helsinki. Written informed consents were obtained from the patients’ guardians. 

### 2.2 Fish Oil and vitamin

Seven Seas fish oil syrup (Seven Seas, Pure Cod Liver Oil, Seven Seas Ltd, UK) was used in the present study. According to the supplement information sheet, every 10 ml Seven Seas consists of 9.2 g of cod liver oil, 828 mg of eicosapentaenoic acid, 736 mg of docosahexaenoic acid, 4000 IU of vitamin A, 400 IU of vitamin D, and 10 IU vitamin E. The fish oil (170 mL per bottle) was given to the patients of group 1 at the beginning of the study for 6 months and at the monthly follow-up visits. Parents were instructed to give 0.5 mL/kg/day Seven Seas once a day to their children. They were asked to bring the bottles back at each follow-up visit. The amount of fish oil remaining in the bottle was checked and a further supply was given monthly.

All children in groups 2 and 3 received 400 units vitamin D and 4000 units vitamin A as recommended by World Health Organization.

### 2.3 Blood samples

Blood samples were collected after aforementioned procedures by venipuncture in vacutainers containing EDTA and immediately placed on melting ice. Plasma was separated not later than 2 hours after sample collection. Half of each blood sample was centrifuged at 1500 ×g for 15 min and the plasma was separated and immediately stored at - 80 C. For determination of 8-*iso*-prostaglandin F2α (iPF2α), 1 μL of butylhydroxytoluene (BHT) 10 mM was added to each 100 μL of the plasma before dividing it into aliquots. Also some of plasma was used to determine ferric reducing antioxidant power (FRAP) value. The second half of each blood sample was centrifuged at 3000 ×g for 10 min for separation of erythrocytes. The red blood cells were washed 3 times with 0.9% normal saline and then lysed with 5 volumes of deionized ice-cold water, distributed into several small vials, and stored at - 80° C for determination of hemoglobin, antioxidant enzymes activities including catalase and erythrocyte glutathione peroxidase (GPX).

### 2.4 Reagents

Drabkin’s reagent, ethylenediaminetetraacetic acid (EDTA), ferric chloride (FeCl3), glutathione (GSH), glutathione reductase (GSSG-R), and sodium azide (NaN3) were purchased from Sigma- Aldrich (St. Louis, MO, http://www.sigma-aldrich.com). In addition, Brij 35, butylhydroxytoluene (BHT), dihydronicotinamide adenine dinucleotide phosphate (NADPH), hydrochloric acid (HCl), hydrogen peroxide (H2O2), methanol, potassium dihydrogen phosphate (KH2PO4), potassium hydroxide (KOH), sodium acetate (CH3COONa), tert-butyl hydroperoxide (TBH), and 2,4,6-Tris(2-pyridyl)-*s*-triazine (tptz) were obtained from Merck Chemicals (Darmstadt, Germany, http://www.merck.de). Also, quercetin was obtained from Acros Organics (Geel, http://www.acros.com). Spectrophotometeric measurements were carried out by a Bio-Tek spectrophotometer (Uvikon XL) and a Bio-Rad microplate reader (680).

### 2.5 Echocardiographic methods

Echocardiographic measurements were performed at the beginning and in the end of the study in groups 1 and 2. Echocardiography was performed with a GE Vivid 3 echocardiographic machine (GE Vingmed, Horten, Norway) using a 3-MHz probe with pulsed Doppler tissue imaging software. All M-mode, two-dimensional, Doppler, and pulsed tissue Doppler echocardiographic studies were conducted, with the patient in left lateral decubitus position by the same qualified cardiologist. Ejection fraction, shortening fraction, and septal and posterior wall thickness in systole and diastole were measured in the left parasternal long axis view. The pulsed Doppler sample volume was placed at the mitral valve and tricuspid tips and three cardiac cycles were recorded from the apical window. Early (E) and late (A) peak velocities (m⁄s) and their ratio were determined to evaluate the diastolic function. Pulsed tissue Doppler images were obtained with the sample volume placed at the lateral corner of the mitral annulus, at the medial (or septal) and tricuspid corner in the apical four-chamber view, and then at the anterior and posterior walls in the parasternal short-axis view. In each region, systolic (S) wave and early diastolic (Ea) as well as late diastolic (Aa) velocities were recorded along with an average of three successive waves. 

### 2.6 Determination of catalase activity of erythrocytes

Erythrocyte catalase activity was spectrophotometrically determined (23). The assay was based on determination of the hydrogen peroxide decomposition in the presence of catalase in erythrocytes by measuring the absorbance change at 240 nm. The final reaction mixture (3.0 mL) contained 2.8 mL of sodium phosphate buffer 50 mM pH 7.2, 100 μL of hemolysate diluted with deionized water (1:3), and 100 μL of hydrogen peroxide 30 mM. The absorbance was determined by a spectrophotometer after 1 min. Proper blank samples were run in parallel. Each unit of catalase activity is defined as the amount of the enzyme required to decompose 1 µmole of hydrogen peroxide (ε = 43.6). For normalization of results, Hemoglobin levels in RBCs lysate measured using the Drabkin's reagent.

### 2.7 Determination of glutathione peroxidase activity in erythrocytes

GPX activity in erythrocytes was measured using the Paglia and Valentine method (24) in which the oxidation of glutathione and subsequent production of GSSG (oxidized glutathione) by GPX enzyme is coupled to the oxidation of NADPH by glutathione reductase (GSSG-R). The oxidation of NADPH is followed spectrophotometrically at 340 nm and the rate of its decay was directly considered proportional to the level of GPX enzyme. The reaction mixture contained 50 μL of the hemolysate diluted (1:1), with Drabkin’s reagent, 2.58 mL of potassium phosphate buffer 50 mM (pH 7, EDTA 5 mM), 100 μL of NADPH 2.9 mM, 6.4 μL GSSG-R 455 U/mL, 10 μL of sodium azide 290 mM, and 100 μL glutathione 58 mM. The reaction was started by adding 100 μL of *tert*-butyl hydroperoxide 17.4 mM. Appropriate Proper blank samples were also run in parallel. The absorbance at 340 nm was measured spectrophotometically every min for 5 min. In addition, the oxidation of NADPH was determined by using a molar extinction coefficient of 6.22 x 103 Cm-1 M-1 at 340 nm. One unit of GPX activity is defined as the amount of the enzyme required for oxidizing 1 µmole of NADPH per minute.

### 2.8 Determination of total antioxidant activity of plasma by FRAP assay

Ferric reducing antioxidant power (FRAP) assay was performed according to the method of Benzie and Strain (25) with minor modifications (26). FRAP solution was prepared by mixing 10 mL of acetate buffer 300 mM, pH 3.6, 1 mL of ferric chloride hexahydrate 20 mM in distilled water, and 1 mL of 2,4,6-*tris *(2-pyridyl)-s-triazine 10 mM in HCl 40 mM. Ten µL of plasma was mixed with 190 µL of FRAP solution in 96-well microplates in duplicate, along with one blank for each plasma sample. The absorbance was measured at 595 nm after 30 min of incubation at room temperature by a microplate reader. FRAP value of the plasma samples were calculated in reference to quercetin, an antioxidant flavonoid, which was tested at a final concentration of 5 µM.

### 2.9 Determination of the total plasma level of 8-iso-prostaglandin F2α (iPF2α)

The level of 8-iso-Prostaglandin F2α (iPF2α), an important index of lipid peroxidation, was determined by an EIA Kit. Briefly, 40 μL of plasma was incubated with 40 μL KOH (15 %) in the dark at 40 C temperature of for 1 hour to hydrolyze the esterified forms of iPF2α. This was then supplemented with 80 µL of ethanol 30% containing 0.01% butyl hydroxytoluene (BHT). After 5 minutes incubation at 4 C, the mixture was centrifuged at 3000 × g for 10 min. The pH of the supernatant fluid was adjusted to 7 by a solution of KH2PO4 1.25 M. The mixture was then diluted by the kit assay buffer (750 µL), and iPF2α level was determined by a commercially available kit. (8-isporostane EIA Kit at www. www.caymanchem.com )

### 2.10 Statistical analysis

The comparisons between the study groups were made by ANOVA one-way analysis of variance. Moreover, Mann–Whitney and Kruskal-Wallis tests were used to compare the non-parametric variables. The data were expressed as mean ± standard deviation. All statistical analyses were performed by SPSS statistical software for Windows (version 16).

## 3. Results

At the beginning of the study, the mean ages of the patients in groups 1, 2, and 3 were 7.5 (range 3-56), 8 (range 5-47), and 7.8 (range 3-58) months, respectively. In addition, the respective mean weight of group 1 at the beginning and at the end of the study was 6.3 ± 5.2 kg and 9.4 ± 6.3 kg (P=0.37). The mean weight of group 2 at the beginning of the study was7.1 ±6.7 kg, and at its end was 8.0 ± 7.8 kg (P= 0.83).Besides, the mean weight of the third group was 8.1 ± 7.3 kg. There were 3 males and 3 females in each group. The mean of weight gain in groups 1 was 2.3 ± 1.3 and in group 2 it was 1.7 ± 1.1 (P=0.40).

### 3.1 Echocardiographic and tissue Doppler parameters

M-Mode echocardiographic parameters including ejection fraction EF, shortening fraction SF, and left ventricle end diastolic dimension were performed at the beginning and at the end of the study in groups 1 and 2 and summarized in Table 1. Doppler indexes of early diastolic (E) wave and atrial contraction (A) wave velocity of Mitral and Tricuspid Valve were also summarized in Table 1.

**Table 1:** M-Mode Echocardiographic Parameters: EF, SF, LVEDD and Doppler Indices of A and E Wave Velocity of Mitral and Tricuspid Valves

variable		Group1	P value within group1	Group2	P value within group2	P value within group
EF	(1)[Table-fn fn5067]	31.63 ± 9.30 %	0.01	45.75 ± 14.14 %	0.35	0. 1
	(2)[Table-fn fn5068]	39.17 ± 13.76 %		40.50 ± 14.03%		
SF	(1)	14.63 ± 4.86 %	0.01	19.17 ± 7.30 %	0.84	0.1
	(2)	23.13 ± 7.93 %		20.00 ±7.23%		
LVEDD	(1)	4.13 ± 0.80 Cm	0.1	4.23 ± 0.68 Cm	0.1	0.1
	(2)	4.14 ± 0.90 Cm		4.43 ±0.83 Cm		
E(MV)	(1)	1.12 ± 0.35 m/s	0.1	0.96 ± 0.26 m/s	0.1	0. 1
	(2)	1.13 ± 0.24 m/s		0.98 ± 0.21 m/s		
A(MV)	(1)	0.84 ± 0.29 m/s	0.89	0.83 ± 0.37 m/s	0.77	0.76
	(2)	0.82 ± 0.21 m/s		0.77 ± 0.34 m/s		
E/A (MV)	(1)	1.38± 0.36	0.23	1.25± 0.30	0.54	0.96
	(2)	1.40± 0.23		1.39 ± 0.45		
E (TV)	(1)	0.78 ± 0.21 m/s	0.63	0.99 ± 0.24 m/s	0.23	1
	(2)	0.85 ± 0.28 m/s		0.85 ± 0.12 m/s		
A (TV)	(1)	0.54 ± 0.10 m/s	0.97	0.80 ± 0.30 m/s	0.72	0.56
	(2)	0.55 ± 0.72 m/s		0.74 ± 0.28 m/s		
E/A(TV)	(1)	1.47± 0.37	0.77	1.33± 0.39	0.65	0.25
	(2)	1.55± 0.56		1.24± 0.29		

(1), Beginning of the study; (2), End of the study

Abbervations: EF, Ejection Fraction; SF, Shortening Fraction; LVEDD, Left Ventricle End Diastolic Dimension; E, Early diastolic; MV, wave velocity of Mitral Valve; A, Atrial Contraction; E/A, E wave velocity to A wave velocity ratio; TV, Tricuspid Valve.

Finally, tissue Doppler indexes of lateral annular velocity of mitral and tricuspid valve as well as septal waves were measured and demonstrated in Table 2.

**Table 2:** Tissue Doppler Indices of Lateral Annular Velocity of Mitral and Tricuspid Valves and Septum

Variables		Group 1	*P value* within group 1	Group 2	*P value* within group 2	*P value* Between groups
Ea Mitral Annulus	(1)[Table-fn fn5070]	6.45 ± 2.76 cm/s	0.09	10.53 ± 4.94 cm/s	0.90	0.59
	(2)[Table-fn fn5071]	9.56 ± 3 cm/s		10.87 ± 4.99cm/s		
Aa Mitral Annulus	(1)	6.45 ± 2.42 cm/s	0.56	9.72 ± 3.08 cm/s	0.60	0.38
	(2)	7.17 ± 1.74 cm/s		8.67 ± 3.66 cm/s		
Sa Mitral Annulus	(1)	5.85 ± 1.70 cm/s	0.88	5.87 ± 1.62 cm/s	0.83	0.69
	(2)	5.73 ± 1 cm/s		6.09 ± 1.92 cm/s		
Ea Tricuspid Annulus	(1)	11.73 ± 4.75 cm/s	0.008	14.37 ± 1.65 cm/s	0.70	0.50
	(2)	16.52 ± 5.17 cm/s		14.89 ± 2.86cm/s		
Aa Tricuspid Annulus	(1)	11.51 ± 4.16 cm/s	*0.01*	10.77 ± 3.14 cm/s	0.89	*0.06*
	(2)	14.94 ± 2.54 cm/s		11.05 ± 3.86 cm/s		
Sa Tricuspid Annulus	(1)	10.11 ± 2.69 cm/	0.75	8.89 ± 2.79 cm/s	0.80	0.22
	(2)	10.67 ± 3.46 cm/s		8.52 ± 2.23 cm/s		
Ea Septum	(1)	7.82 ± 3.07cm/s	*0.002*	10.18 ± 2.48 cm/s	0.94	0.73
	(2)	10.65 ± 3.11 cm/s		10.08 ± 2.61 cm/s		
Aa Septum	(1)	7.44 ± 2.13 cm/s	0.83	7.17 ± 2.39 cm/s	0.51	0.69
	(2)	7.73 ± 2.65 cm/s		8.63 ± 4.69 cm/s		
Sa Septum	(1)	5.10 ± 0.81cm/s	*0.01*	5.34 ± 2.07 cm/s	0.89	0.14
	(2)	6.21 ± 0.50 cm/s		5.20 ± 1.47 cm/s		

(1), Beginning of the study; (2), End of the study

Abbervations: Aa,Tissue Doppler atrial wave velocity; E a , tissue Doppler early diastolic wave velocity; Sa: Systolic Wave velocity

### 3.2 Biomarkers of oxidative stress

Activities of antioxidant enzymes, including catalase and GPX were measured in erythrocytes, while FRAP and iPF2α were determined in plasma of the subjects of three groups at the end of the study (Table 3). Catalase and GPX activities in group 1 (n-3 PUFA plus anti-failure therapy) was decreased compared to group 2 (anti-failure therapy) and group 3 (normal control). However, these changes were not statistically significant. FRAP values did not show any considerable change among the 3 groups. Plasma iPF2α levels were increased in group 1 compared to groups 2 and 3, which were not statistically significant (Table 3).

**Table 3:** Comparison of Oxidative Stress Biomarkers in Three Groups

	Group 1[Table-fn fn5072]	Group 2[Table-fn fn5073]	Group 3
Catalase (U/g Hb)	1189.2 ± 620.9	1841.9 ± 641.5	2043.6 ± 1350.6
Glutathione peroxidase (U/g Hb)	11.8 ± 1.4	16.1 ± 3.3	16.5 ± 7.7
FRAP (µEq Q/L)	118.0 ± 37.5	129.7 ± 62.6	115.3 ± 31.6
8-iso-Prostaglandin F_2∝_	21.3 ± 10.8	15.0 ± 5.3	13.5 ± 4.6

Group 1: consisted of pediatric patients diagnosed with dilated cardiomyopathy (DCMP), who received omega-3 polyunsaturated fatty acids (PUFA) and anti-failure therapy; Group 2: were DCMP patients who only received anti-failure therapy; Group 3: consisted of normal individuals

Abbervations: U/g, Unit/gram hemoglobin;μEq Q/L, μmole equivalent of quercetin/L

## 4. Discussion

Echocardiographic findings in group 1 showed improvement in several parameters after fish oil supplementation compared to before treatment, whereas the group 2 which only received anti failure therapy did not show any significant change after treatment. Oxidative stress biomarkers did not show any significant alteration in any of the 3 groups.

The main therapeutic goal in children with DCMP is to avoid heart transplant and early death. These goals can be achieved by early diagnosis, risk assessment, and application of new therapies, including dietary modification, anticongestive agents, angiotensin-converting enzyme inhibitors, and antiarrhythmic agents (4) . In addition, fish oil is known to have beneficial effects on cardiovascular diseases in adults (20, 21, 27); however, few studies have addressed the effect of fish oil on children with DCMP (28).

Our study showed that fish oil treatment in group 1 increased left ventricular ejection fraction (EF) compared to the same patients before treatment. Olgar and colleagues (28) conducted a study on the effect of fish oil on idiopathic DCMP in children and suggested that fish oil supplementation led to accelerated improvement of left ventricular function. In their study, left ventricular EF and left ventricular SF were significantly increased by 8.44 ± 3.80 % and 6.04 ± 4.86 %, respectively, while left ventricular end- diastolic dimension was decreased by 4.36 ± 4.86 mm (P<0.05). This finding suggests that fish oil may accelerate the improvement in left ventricular function, which might be affected by antiatherogenic, antithrombotic, anti-inflammatory, and antiarrhythmic effects of n-3 PUFA. In addition, it has been demonstrated that fish oil decreases the mortality rate, creatine kinase levels, myocardial lipid peroxides, and glutathione levels in experimental myocardial infarction induced by isopreterenol (28). However, in study by Rizos EC et al. in Greece, omega-3 supplementation was not associated with reduction in the risk of cardiovascular mortality, sudden death, myocardial infarction, or stroke (29).

In our study, the lateral tricuspid valve annular velocities in tissue Doppler (S wave, Ea and Aa waves) were measured and showed a significant increase in Ea and Aa waves in the DCMP patients during the follow up (P<0.05); however, they did not show any significant changes in group 2 patients who did not receive fish oil which indicates that n-3 could improve the diastolic function. The same significant improvement was seen in Ea wave and S wave velocities of septum in group 1. S wave velocity of septum was significantly increased in group 1, which might favour the increased systolic function in the fish oil-treated group. Our results showed that antioxidant enzymes, including catalase and GPX in erythrocytes of DCMP patients treated with n-3 PUFA (group 1) were slightly decreased compared to DCMP patients treated only with anti-failure therapy (group 2) and also to normal individuals of the same age (group 3), however these changes did not reach the level of statistical significance. Surprisingly, plasma iPF2α concentrations, that reflect oxidative modification of arachidonic acid were slightly increased without any statistical significance in n-3 PUFA-treated compared to those receiving anti-failure therapy and normal subjects, although these changes were not statistically significant. FRAP values that reflect the total antioxidant capacity of plasma were similar in all 3 groups. To our knowledge, there is no report on the effect of n-3 PUFA on oxidative stress biomarkers in children with cardiomyopathy. Some earlier studies had shown a reduction in oxidative stress biomarkers in adult individuals supplemented with n-3 PUFA (30, 31); however, several more recent investigations have not supported this notion (32).

Studies on hypercholesterolemic (33) and normocholesterolemic (34) subjects have shown that n-3 PUFA supplementation increases plasma malondialdehyde (MDA), a marker of lipid peroxidation. Also exercise-induced increase of MDA has been greater in judo athletes supplemented with n-3 PUFA compared to placebo (35). A large dietary intervention study in subjects with metabolic syndrome (LIPGENE study) (36), and also another research performed on healthy women (37) supplemented with n-3 PUFA, did not show any change in urinary levels of iPF2α. Similarly, acute supplementation of fish oil had no effect on lipid hydroperoxides and oxidized low density lipoprotein in obese men (37). The report of Carrepeiro and colleagues (33) shows that n-3 PUFA reduce catalase expression in women , while other reports on chronic renal failure patients with dyslipidemia showed increased serum Catalase, superoxide dismutase and glutathione peroxidase activities (38).

Our results were in agreement with findings of KANWU study on total antioxidant capacity performed on 162 participants, (31) who received n-3 PUFA for 3 months, as well those reported on obese men (37), receiving an acute supplementation of n3-PUFA, where there were no significant changes in the plasma total antioxidant activity.

## 5. Conclusion

The results of the present study showed that some echocardiographic parameters, such as left ventricular EF, SF, Ea, Aa waves of lateral annulus of tricuspid valve and Ea wave and S wave of septum were significantly improved in omega-3 fish oil receiving group. However, omega-3 had no statistically significant effect on oxidative stress biomarkers.
